# Clinical features and comprehensive treatment of persistent corneal epithelial dysfunction after cataract surgery

**DOI:** 10.1186/s12886-024-03466-x

**Published:** 2024-04-26

**Authors:** Xianwen Xiao, Yuan Lin, Xie Fang, Zhiwen Xie, Shunrong Luo, Huping Wu

**Affiliations:** 1 Xiamen Eye Center and Eye Institute of Xiamen University, Xiamen, China; 2 Xiamen Clinical Research Center for Eye Diseases, Xiamen, Fujian China; 3 Xiamen Key Laboratory of Ophthalmology, Xiamen, Fujian China; 4 Fujian Key Laboratory of Corneal & Ocular Surface Diseases, Xiamen, Fujian China; 5 Xiamen Key Laboratory of Corneal & Ocular Surface Diseases, Xiamen, Fujian China; 6 Translational Medicine Institute of Xiamen Eye Center of Xiamen University, Xiamen, Fujian China

**Keywords:** Persistent corneal epithelium dysfunction, Intense pulsed light, Meibomian gland dysfunction, Cataract surgery, Tobramycin/dexamethasone

## Abstract

**Objective:**

Evaluation of clinical efficacy and safety of tobramycin/dexamethasone eye ointment in treating persistent corneal epithelial dysfunction (PED) after cataract surgery.

**Methods:**

26 cases diagnosed as PED after cataract surgery accept the tobramycin/dexamethasone ophthalmic ointment and intense pulse light treatment in the Xiamen University of Xiamen eye center between September 2016 and April 2022 were retrospectively analyzed, mainly including clinical manifestations, characteristics of morphological changes imaged by in vivo confocal microscopy, meibomian glands infrared photography, lipid layer thickness (LLT), management and therapeutic effects.

**Results:**

There were 26 eyes, include 8(35%) males and 15(65%) females with an average age of 69.6 ± 5.2 years(50 to 78 years). The mean hospitalization time was (18.4 ± 7.5) days after cataract surgery. Twenty patients had meibomian gland dysfunction. Infrared photography revealed varying loss in the meibomian glands, with a mean score of 3.8 ± 1.2 for gland loss. The mean LLT was 61.6 ± 8.4 nm. After treatment, 20 patients were cured, and 3 received amniotic membrane transplantation. After treatment, the uncorrected visual acuity (UCVA) and best-corrected vision activity (BCVA) improved (*P* < 0.001), and there was no significant difference in intraocular pressure (IOP) before and after treatment (*P* > 0.05).

**Conclusions:**

The early manifestation of PED after surgery is punctate staining of the corneal epithelium. Tobramycin and dexamethasone eye ointment bandages have a good repair effect. The meibomian gland massage combined with intense pulse light treatment can effectively shorten the course of the disease.

## Introduction

Persistent corneal epithelial dysfunction (PED) is the pathological state of persistent corneal epithelial defect caused by an abnormal repair process of corneal epithelial damage caused by various factors under the premise of a regular source of limbal stem cells [1]. Impaired PED can lead to epithelial lesions, such as post-operative edema and shedding. Major risk factors for inflammation after cataract surgery include diabetes, tear film problems, meibomian gland dysfunction (MGD), and nonsteroidal anti-inflammatory drug use [2, 3]. Inflammation can result in severe damage and hinder repair in individuals with diabetes. Proper and prompt identification and treatment of corneal ulcers is of utmost importance. Ignoring this condition can result in the onset of bacterial or viral infections and adverse effects on a patient’s surgical outcomes and postoperative vision restoration. Patients undergoing cataract surgery may experience corneal epithelial dysfunction, leading to discomfort, foreign body sensations, and hindered visual function recovery. Research by Nishida et al. revealed that approximately 5.2% of cataract patients experienced spontaneous epithelial erosion, with 63.4% developing epithelial defects. This complication is linked to the dysfunction of basal cells’ focal adhesion [4]. Al-Hinai found corneal epithelial cell connectivity dysfunction and increased epithelial permeability in patients undergoing cataract surgery [5].

The ocular drug products of tobramycin dexamethasone have utility in treating various clinical manifestations of inflammatory diseases with underlying infectious ocular surface diseases [6]. 0.3% tobramycin/dexamethasone 0.1% significantly reduced ocular inflammation signs (blepharitis, secretions, conjunctivitis) and inflammation score [7]. Previous studies have shown the reliable safety and efficacy of tobramycin dexamethasone eye drops for inflammation caused after cataract surgery [8]. There is currently no established protocol for treating corneal epithelial dysfunction following cataract surgery. Various options are available, such as utilizing preservative-free artificial tears, personalized serum, wearing a corneal bandage patch, or resorting to amniotic membrane transplantation (AMT) in cases where less invasive treatments prove ineffective [9–11]. Our study aims to analyze the safety and efficacy of combining tobramycin dexamethasone eye dressing with autologous serum and intense pulsed light (IPL) for treating corneal epithelial dysfunction post-cataract surgery.

## Method

### Patients

The research followed the ethical principles outlined in the Declaration of Helsinki. The Human Ethics Committee of Xiamen University, affiliated with Xiamen Eye Center, reviewed and approved the studies involving human participants. All patients provided written informed consent before surgery, including their agreement to use their data in future teaching and research at the institution. Clinical data of 23 patients with 26 eyes from September 2016 to April 2022, including eight males and fifteen females, aged 50 to 78, mean (69.6 ± 5.2) years. All patients underwent cataract surgery with a phacoemulsification procedure.

### Diagnosis, inclusion and exclusion criteria

The inclusion criteria all met the diagnostic criteria for corneal epithelial dysfunction: (1) Eye symptoms. (2) Characteristic injury of corneal epithelium: diffuse punctate deletion, erosion, epithelial defect. 3 The anterior segment optical coherence tomography suggests the absence of the corneal epithelial layer. 4 Decreased corneal sensitivity or in vivo confocal microscopy indicates abnormal morphology and density of corneal nerve fibers. The occurrence of either item 2 or item 3 can be diagnosed as corneal epithelial injury. Item 4 only indicates that the corneal nerve function is abnormal and needs to be judged with other auxiliary tests.

The exclusion criteria were as follows: patients with coexisting ocular surface diseases, including corneal penetrating keratoplasty lesions, neurotrophic corneal ulcers, chronic graft-versus-host disease, as well as conjunctival keratosis, and exposed corneal lesions, will be excluded. Traumatic PEDs will also be excluded.

### Clinical evaluation

The observation period of all patients before starting treatment was 14 days, higher than 14 days was regarded as progressing to PED and adding the patient to our hospital to start treatment. All patients underwent a thorough examination of their systemic disease history, including the onset and recovery time. The examination included uncorrected visual acuity (UCVA) and best-corrected visual acuity (BCVA) measurement, intraocular pressure (IOP), corneal fluorescein sodium staining, meibomian glands’ infrared photography, tear film lipid layer thickness, and in vivo confocal microscopy.

At every visit, visual acuity and slit-lamp examination were conducted to document disease activity, the extent of corneal involvement, and signs of resolution or deterioration. Healing was defined as the absence of ulcer progression and filling of ulcer craters without fluorescein staining. Once signs of disease remission became apparent, local steroids were gradually tapered over the next 2–3 weeks and then discontinued. All patients were monitored for evidence of recurrence for at least three months after medication discontinuation. Follow-up appointments were scheduled at weeks 1 and 2 and at the end of months 1 and 3.

The BCVA method uses statistical analysis to measure visual acuity using the Snellen chart. The results are then converted to Logarithm of Minimum Angle of Resolution (LogMAR) units. We excluded any ocular condition that could affect BCVA were excluded from the analysis regarding BCVA [12].

### Corneal fluorescein staining(CFS)

The patients ocular surface soaked in fluorescein. Then, patient blinked several times, after 3 min CFS was evaluated by a slit-lamp microscope illuminated with cobalt blue to evaluate localized corneal and conjunctival epithelial desiccation areas. Staining was recorded using the modified Oxford grading scheme [13].

### Infrared photography of the meibomian glands

The Keratograph 5 M ocular surface analyzer (OCULUS Germany) was used to analyze, during the evaluation, the patient faced a 5 M corneal topographer with the chin supported under proper support. Then, the patient sat in front of the 5 M anterior corneal camera using the Meibo-Scan procedure and scored: 0 points = no loss; 1 score = less than 1 / 3 of the total amount of meibomian glands; 2 points = 1 / 3 to 2 / 3 of the total amount of glands; 3 scores = more than 2 / 3 of the total amount of meibomian glands. The total loss of the upper and lower eyelid glands is 0–6 [14].

### Lipid layer thickness (LLT)

The mean LLT measurements were taken using a lipid view interferometer (manufactured by Tears Science, Inc. in Morrisville, North Carolina, USA). The patient was asked to stay still during the test while a 20-second video of their tear film interferometry image was recorded. The unit of measurement used was the interferometric color unit (ICU), which corresponds to approximately 1 nm with an ICU. If the LLT were more significant than 100 nm, the laser map interferometer would show the maximum value of 100 nm.

### In vivo confocal microscopy (IVCM)

A slit lamp microscope (BQ900IM900) and its photographic device (Haag-Streit, Switzerland) were used to take images of the front part of the eye. The IVCM (HRT III/Rostock) was used to examine the cornea. To prepare for imaging, 0.5% proparacaine was applied to the conjunctival capsule three times, and a gel (Alcon, Fort Worth, TX) was placed between the lens surface (made of polymethacrylate or PMMP)—the lens contact cap to serve as the imaging medium. A 670 nm semiconductor laser was used as the excitation light source, and manual rotation adjustment was made to get a clear image. The objective was a 60 intrusive lens with 800x magnification, a scan area of 400 μm², and a picture pixel of 384 × 384. The central cornea was used as a reference point, and the different layers of the cornea, such as the corneal epithelial layer, Bowman’s layer, stroma, Descemet’s membrane, and endothelial layer, were examined vertically.

### Treatment strategies

Patients who were hospitalized stopped using their original medications after excluding microbial reasons and contraindications. They then underwent vital pulse laser treatment and targeted treatment of eyelid gland hot compress massage. Tobramycin dexamethasone eye ointment was pressurized and bandaged overnight, autologous serum was administered four times a day, and artificial tears without preservatives were used four times daily.

Individual cases required AMT, and the surgical techniques were as follows: after peribulbar or local anesthesia, used a micro sponge to clear the base of epithelial defects or interstitial ulcers and removed poorly adhered epithelial cells around the defects or ulcers. Placed the amniotic membrane covering the surface of the cornea. After each AMT, used bandaged contact lenses [15].

### Data analysis

mean ± standard deviation and non-normal distributed data will be described by median (25%∼75% percentile, interquartile range, IQR). Appropriate statistical methods were selected based on the normal distribution and homogeneity of data variances. For measurement data, if not meeting normality and uneven variance, the Wilcoxon non-parametric method was used, and a paired t-test was used for before and after comparison of the same groups. All data analyses were performed using SPSS 25 version software (IBM, Armonk, New York) and Excel software (Microsoft, Redmond, Washington). *P*-values of less than 0.05 were considered significant.

## Result

Among the 23 patients (26 eyes), the mean visit was (18.4 ± 7.5) days after cataract surgery. Eight males (35%) males and fifteen females (65%), with a mean age of 69.6 ± 5.2 years (50–78 years). Seven patients had diabetes, and one patient had rheumatoid disease. Twenty patients had associated MGD. The infrared photography of the meibomian glands showed different degrees of gland loss, and the average score of the lower gland loss was (3.8 ± 1.2). The clinical manifestations of corneal epithelial dysfunction after cataract surgery are increased corneal epithelial permeability, localized epithelial edema, corneal epithelial spot staining, corneal epithelial defect and ulceration, corneal stromal edema, and posterior elastic laminae.

**Fig. 1:**
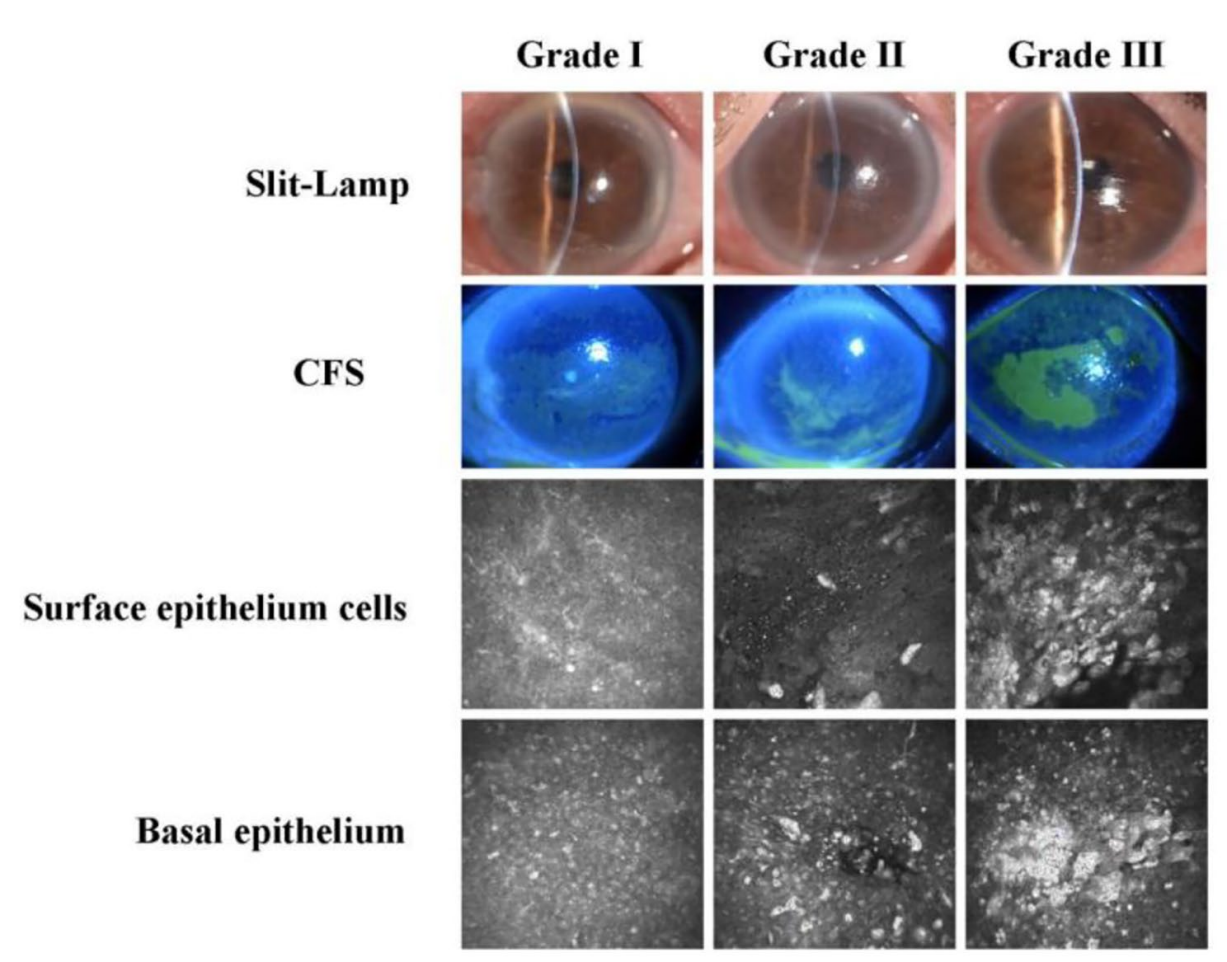
After cataract surgery, we have classified the PED into three grades based on slit lamp and fluorescent staining. By combining clinical and IVCM characteristics, we believe that the ocular surface state of PED after cataract surgery is a dynamic process between dry eye and corneal ulcer. This process is influenced by persistent epithelial dysfunction

The degree of corneal epithelial damage (Fig. 1) was divided with the help of corneal fluorescein staining: Grade I: punctate loss of corneal epithelium. Corneatopathy features include a diffuse punctate or point-tufted opacification of the corneal epithelium. Grade II: Large range of loss of corneal epithelium erosion fusion into pieces, false dendritic opacity. Grade III: Large corneal epithelial defect or corneal stromal ulcer formation. A laser confocal microscopic examination of the cornea found that the corneal epithelium was scattered in the defect, mixed with small round inflammatory particles, the enhancement of the corneal epithelial layer, and mixed with dendritic cells. The peripheral subepithelial nerve fibers were thinner and decreased in density. The superficial cells of the matrix were activated, with no apparent inflammatory cell infiltration, and granular inflammatory high reflection was seen on the endothelial surface of the cornea in some patients—enhanced reflection in the corneal basal cell nuclei. The corneal sub-basal nerve plexus morphology was disordered and compact (Fig. 1).

The Lipiview ocular surface interferometer was used to measure the thickness of the lipid layer before and after treatment. The mean LLT was (61.6 ± 8.4) nm before and (63.4 ± 6.1) nm after treatment that changes were not statistically significant (*P* > 0.05). Out of the 23 patients treated, all showed clinical improvement and complete repair of the corneal epithelium. The average time for epithelial repair was (5.1 ± 2.1) days, and the patient’s visual acuity improved to varying degrees. The UCVA improved from LogMAR (1.18 ± 3.45) to LogMAR (0.52 ± 0.27)(*P* < 0.001), and the BCVA improved from LogMAR (1.03 ± 2.82) to LogMAR (0.31 ± 0.19)(*P* < 0.001). The pre-treatment IOP was (12.3 ± 5.3) mmHg, which did not change significantly after treatment (*P* > 0.05) (Table 1). Three patients showed a slight improvement in the corneal epithelium after seven days of treatment with tobramycin dexamethasone eye dressing but still had a significant sheet defect. These patients underwent AMT, which resulted in complete repair of the corneal epithelium after amniotic lysis about one month later.

**Table 1:** Changes in visual acuity and intraocular pressure before and after treatment

	UCVA(LogMAR)	*P*	BCVA(LogMAR)	*P*	IOP	*P*
Before treatment	1.18 ± 3.45	< 0.001	1.03 ± 2.82	< 0.001	12.3 ± 5.3	> 0.05
After treatment	0.52 ± 0.27		0.31 ± 0.19		13.1 ± 4.9	

UCVA, Uncorrected visual acuity; BCVA, Best Corrected Visual Acuity; IOP, intraocular pressure

## Discussion

**Fig. 2:**
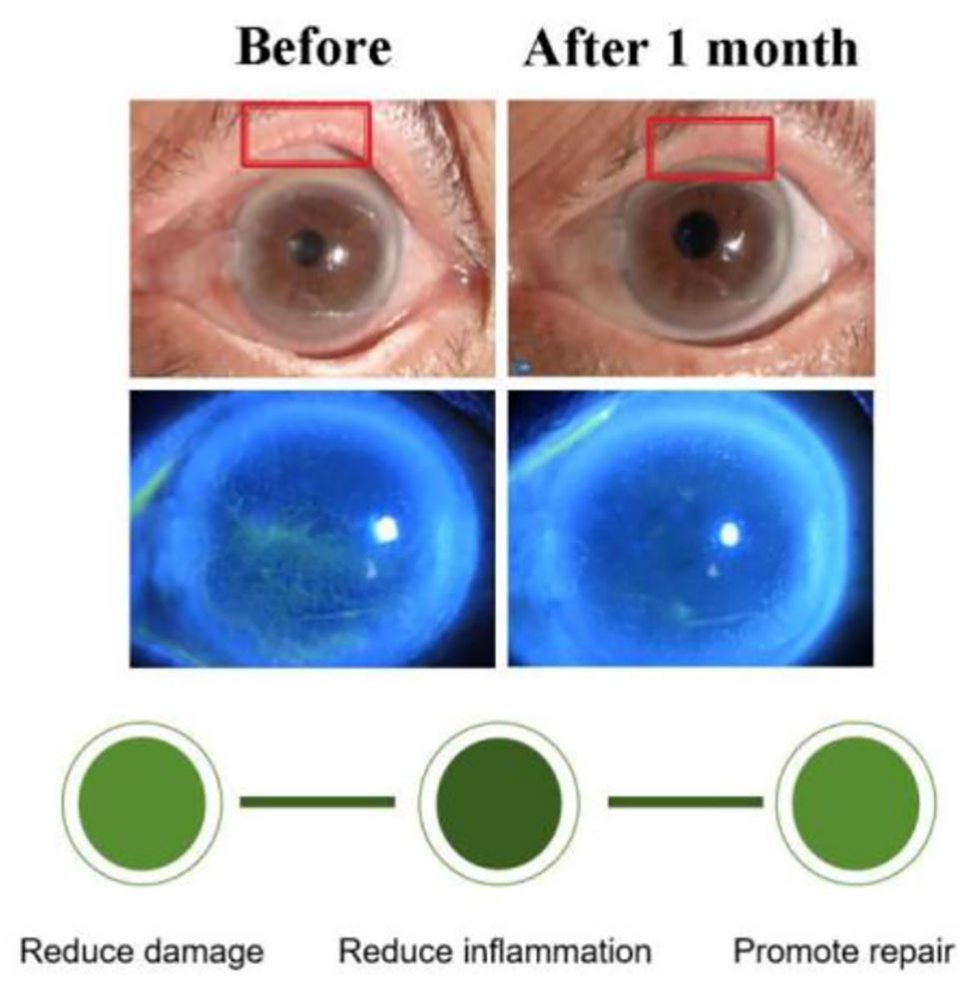
We used a comprehensive treatment approach that combined tobramycin dexamethasone eye ointment dressing with meibomian gland management to treat PED. During the treatment, we noticed improvements in the eyelid margin condition, and the corneal epithelium repair was successful. This comprehensive treatment, which included ointment dressing and meibomian gland management, reduced the stress of the inflammatory response during the treatment of PED and helped speed up the treatment process while consolidating the treatment results

Restoration of corneal function after ocular surgery is closely related to repairing corneal epithelial cells and regeneration. The leading causes of corneal epithelial dysfunction after cataract removal are epithelial cell hyperplasia, adhesion ability, and connection dysfunction [16]. The occurrence of PED after cataract surgery requires consideration of multiple factors. The literature shows that longer surgical time, longer absolute and effective phaco time, higher average ultrasound power, and higher cataract density are significantly associated with endothelial cell loss and trigger corneal epithelial toxicity during and after surgery [17–19]. The preoperative corneal epithelial status including epithelial basement membrane dystrophy [20], dry eye [21], and recurrent corneal erosion [22] may affect the occurrence of PED after cataract surgery. The previous treatment strategy for PED management after cataract surgery has been to reduce injury and promote repair. However, combining medication and eyelid treatment to alleviate ocular surface inflammation may be effective in the treatment of epithelial sheet defects. In this study, tobramycin/dexamethasone eye dressing combined with autologous serum and the intensive treatment of the meibomian gland reduced the underlying inflammatory state of the ocular surface may be an ideal mode for the comprehensive management of the ocular surface in the promotion of epithelial repair. (Fig. 2).

In this study, it was found that some patients received chronic antiviral therapy, including fluoroquinolone or aminoglycoside antibiotics, gancyclovir gel, or acyclovir eye drops, on average 18.4 ± 7.5 days after cataract surgery in other hospitals. It is worth noting that long-term use of antibiotic eye drops can cause adverse drug reactions. Additionally, the common fluoroquinolones associated with ofloxacin may promote the expression of matrix metalloproteinases, resulting in keratopathy [23]. The preservative benzalkonium chloride in eye drops can reduce the proliferation and viability of corneal cells, damage the epithelial barrier, and delay wound healing [24]. Prolonged use of eye drops can cause corneal epithelial dysfunction, leading to tear accumulation and direct damage to the cornea. In our study, IVCM test show inflammation, nerve fiber thinning, activated superficial cells, and increased dendritic cells in people with compromised corneal barriers.

The characteristics of dry eyes after cataract surgery in MGD patients differ from those in ordinary cataract patients. Surgical factors cause changes to the ocular surface in the early postoperative period, and the damage to epithelial function in the late postoperative period is mainly related to the inflammation of the meibomian gland and eyelid [25]. In our study, infrared absence of gland, with the mean score of (3.8 ± 1.2). This suggests that patients with MGD before cataract surgery are more likely to develop corneal epithelial dysfunction after surgery. The study found that the total number of bacteria on the ocular surface increased in most MGD patients, among which the bacteria were significantly more than in ordinary people [26]. When the meibomian gland becomes inflamed, it triggers the production of lipids by bacteria, which can harm the epithelial cells, cause instability in the tear membrane, and obstruct the gland duct. The treatment of MGD demands anti-inflammatory therapy. Glucocorticoids, such as dexamethasone, possess anti-inflammatory properties that can alleviate swelling, prevent blood vessel formation, deter white blood cell movement, prevent excessive fibrous tissue growth, and reduce collagen deposition [27]. The tobramycin dexamethasone eye ointment dressing is a versatile solution that lubricates and protects the eye surface, minimizing the impact of corneal friction. Additionally, it effectively alleviates dry eye symptoms and shields the tear film from damage. This eye ointment, containing antibiotics and hormones, is also beneficial in treating chronic blepharitis and MGD lead to corneal epithelial damage [28]. However, Antibiotic treatment are not suitable for antiviral therapy, and personalized treatment plans are needed for different situations. The corneal epithelium notably expresses glucocorticoid receptors, which can potentially enhance the expression of cell membrane tight junction protein-1, thus directly improving barrier function and wound healing [29].

When used with tobramycin dexamethasone eye ointment, IPL treatment, and meibomian gland massage can effectively reduce inflammation and accelerate the healing of corneal epithelial cells for patients suffering from MGD. This combination therapy produces complete epithelial repair in a mere 5.1 ± 2.1 days on average. The advanced laser technology can soften eyelid fat and impede the circulation of small blood vessels around the meibomian gland, inhibiting the release of inflammatory mediators and reducing inflammation [30]. We believe that meibomian gland massage can promote conjunctiva, eyelid capillary relaxation, conjunctiva, eyelid lymph, blood circulation, restore meibomian normal or benign secretion function, and eyelid gland opening after drug penetration and absorption, improve local drug concentration, strengthen treatment effect, combined with tobramycin dexamethasone ointment has a synergistic effect.

Although there were no serious complications during our treatment. Research has shown that local use of ointment after cataract surgery can enter the anterior chamber, leading to severe uveitis [31]. Due to the fact that eye ointment entering the anterior chamber can even cause toxic anterior segment syndrome [32], more caution and detailed follow-up may be required for the use of ointment in incomplete corneal incisions [33, 34]. In addition, topical glucocorticoids used for 4–6 weeks may increase IOP in 5% of patients [35]. There was no significant difference in IOP contrast before and after treatment, which may be related to our short use time, and the corneal epithelial repair time was only (5.1 ± 2.1) days. Eye gel can prolong the residence time of drugs on the ocular surface, shorten the time when the corneal epithelial cells are directly exposed to the air, and protect the newer epithelium. In our study, infrared absence of gland, with the mean score of (3.8 ± 1.2). In addition, we believe that in this study, combined eyelid management can shorten the treatment course, reduce drug dosage, and reduce risk.

Three patients who suffered from a corneal defect were administered tobramycin dexamethasone eye patches for seven days, but regrettably, surgery remained necessary because the repair of the corneal epithelium did not improve. However, the corneal epithelium had fully healed a month after undergoing an amniotic graft. The amniotic membrane is an outstanding basement membrane that facilitates the growth of epithelial cells, prevents apoptosis, and strengthens adhesion and differentiation. Furthermore, it can regulate inflammation and resist keratolysis [36].

One of the primary limitations of this study is that it is retrospective in nature, which can result in selection bias. the data was obtained from a tertiary referral center, which means that there may be limited data volumes available. Another limitation is that we lack a comparable control group, which would affect the compariosn of the effectiveness of the treatment method used in this study.

## Conclusion

A recent study was conducted on treating Posterior Epithelial Defect (PED) following cataract surgery. The results indicate that mitigating inflammatory reactions can restore the ocular surface to a “Cold” state. Applying tobramycin dexamethasone eye ointment dressing can reduce stress on the ocular surface, providing a foundation for subsequent epithelial repair. Proper management of the meibomian gland is also essential in the comprehensive treatment of PED after cataract surgery. We recommend expanding the study sample and the observation period to understand better the effectiveness of the tobramycin dexamethasone eye dressing combination.

## Data Availability

The data presented in this study are included in the article. The data are not publicly available due to restrictions that apply to the availability of the data (e.g., privacy or ethical). Datasets from this study may be available upon request from the corresponding author and provided upon approval from the sponsor and in accordance with data privacy and ethical provisions.

## Abbreviations

PED: Persistent corneal epithelium dysfunction
IPL: Intense pulsed light
MGD: Meibomian gland dysfunction
UCVA: Uncorrected visual acuity
BCVA: Best Corrected Visual Acuity
IOP: Intraocular pressure
IVCM: In Vivo Confocal Microscopy
AMT: Amniotic membrane transplantation
LLT: Lipid layer thickness
